# Supplementary material for: Soloxolone Methyl Reduces the Stimulatory Effect of Leptin on the Aggressive Phenotype of Murine Neuro2a Neuroblastoma Cells via the MAPK/ERK1/2 Pathway
Source: Pharmaceuticals (Basel). 2023 Sep 27;16(10):1369. doi: 10.3390/ph16101369 (PMC10610011; doi:10.3390/ph16101369)
Supplement: Supplementary file 1 [file pharmaceuticals-16-01369-s001.zip › pharmaceuticals-2595735-supplementary.pdf]

Article

Soloxolone Methyl Reduces the Stimulatory Effect of Leptin on the Aggressive Phenotype of Murine Neuro2a Neuroblastoma Cells via the MAPK/ERK1/2 Pathway

Kirill V. Odarenko [1], Oksana V. Salomatina [1,2], Ivan V. Chernikov [1], Nariman F. Salakhutdinov [2], Marina A. Zenkova [1] and Andrey V. Markov [1,*]

[1] Institute of Chemical Biology and Fundamental Medicine, Siberian Branch of the Russian Academy of Sciences, 630090 Novosibirsk, Russia; k.odarenko@yandex.ru (K.V.O.); ana@nioch.nsc.ru (O.V.S.); chernikovivanv@gmail.com (I.V.C.); marzen@niboch.nsc.ru (M.A.Z.)
[2] N.N. Vorozhtsov Novosibirsk Institute of Organic Chemistry, Siberian Branch of the Russian Academy of Sciences, 630090 Novosibirsk, Russia; anvar@nioch.nsc.ru
* Correspondence: andmrkv@gmail.com; Tel.: +7-383-363-51-61

Supplementary material

Table S1. The primers used in the study

Gene	Type	Sequence
Itga1	Forward	5'-CTAACCAGATTGTCATCCCTC-3'
	Reverse	5'-CCAGCGATATAGAGCACATC-3'
Itgb1	Forward	5'-CTGGAGAATGTGAAAAGTCTTG-3'
	Reverse	5'-GGGTTGTGCTAATATACGGC-3'
L1cam	Forward	5'-GAGGTTTCAGGGCATCTATC-3'
	Reverse	5'-GGTTTTACAGTCTCCTTCGG-3'
Ncam1	Forward	5'-GACACTATCTGGTCAAGTACAG-3'
	Reverse	5'-CACATAGACTTCATACTCTGCG-3'
Cdh2	Forward	5'-CCTCCAGAGTTTACTGCCATG-3'
	Reverse	5'-ACCACTGATTCTGTATGCCG-3'
Vim	Forward	5'-CTCCTACGATTCACAGCCAC-3'
	Reverse	5'-GAGCCACCGAACATCCTG-3'
Gapdh	Forward	5'-AAGAGAGGCCCTATCCCAAC-3'
	Reverse	5'-GCAGCGAACTTTATTGATGG-3'

Table S2. The parameters of the molecular docking simulations.

Protein target	PDB ID	Grid box					
		Coordinates			Size		
		x	y	z	x	y	z
JAK2	6VGL	24.400	13.420	53.879	14	18	16
SHP-2	7JVM	20.068	-23.098	27.019	16	10	16
SOS	4NYI	37.466	30.416	22.908	16	14	12
H-RAS	6ZJO	-21.211	40.151	23.363	14	14	14
c-Raf	3OMV	6.360	16.358	33.441	14	18	14
MEK1	7JUR	-55.347	76.653	12.309	16	22	16
ERK2	1TVO	6.416	-4.156	16.954	14	16	14

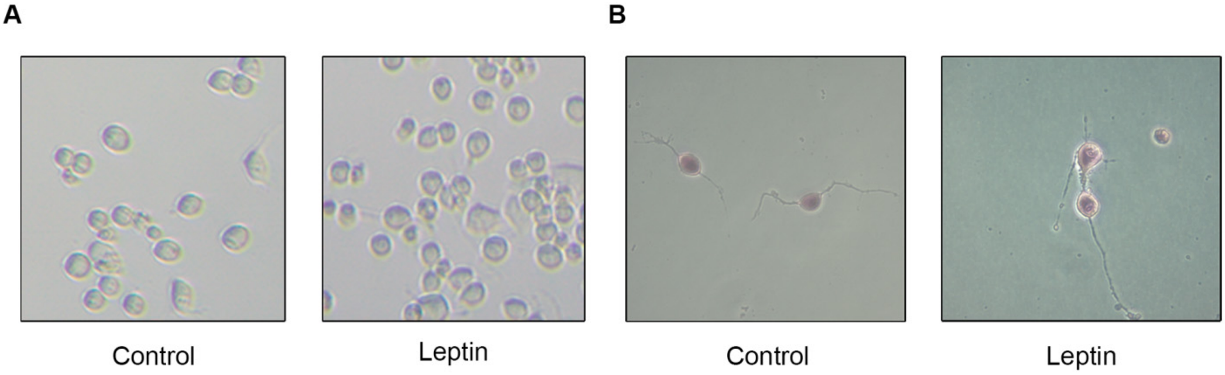

Figure S1. Evaluation of Neuro2a cell morphology after 48-h incubation with leptin (400 ng/ml). (A) Cell size was assessed at ×100 magnification. (B) Neuritogenesis was visualized after crystal violet staining. ×100 magnification.

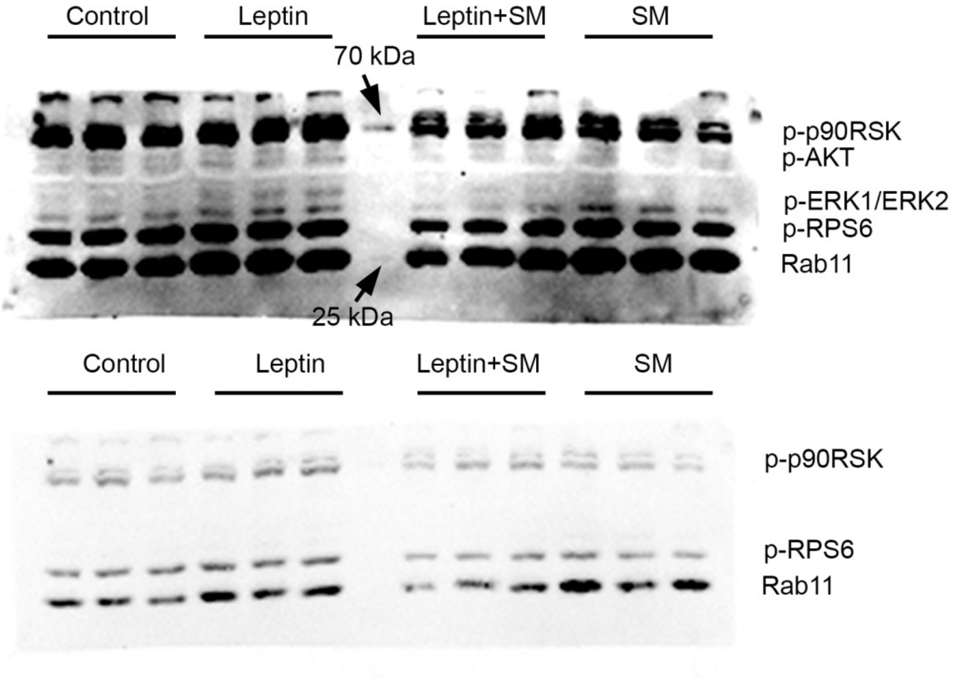

Figure S2. Levels of phosphorylated p90RSK and ERK1/ERK2 in leptin-stimulated Neuro2a cells treated with SM. Neuro2a cells were induced with SM (0.1 μM) for 1 hour and then treated with leptin (400 ng/ml) for 30 minutes. The top and bottom images represent the same blot exposed for a longer and shorter time, respectively.
